# Effects of *Arnica* Phytotherapeutic and Homeopathic Formulations on Traumatic Injuries and Inflammatory Conditions: A Systematic Review

**DOI:** 10.3390/plants13213112

**Published:** 2024-11-04

**Authors:** Claudia-Crina Toma, Mariangela Marrelli, Monica Puticiu, Filomena Conforti, Giancarlo Statti

**Affiliations:** 1 Department of Pharmacognosy, Faculty of Pharmacy, Western University “Vasile Goldiș” of Arad, L. Rebreanu Street, No. 87, 310048 Arad, Romania; claudiatoma2004@yahoo.com; 2 Department of Pharmacy, Health and Nutritional Sciences, University of Calabria, 87036 Cosenza, Italy; filomena.conforti@unical.it (F.C.); giancarlo.statti@unical.it (G.S.); 3 Emergency Department, Arad County Hospital, Faculty of Medicine, Western University “Vasile Goldiș” of Arad, 86 Rebreanu, 310414 Arad, Romania; puticiu.monica@uvvg.ro

**Keywords:** *Arnica*, bruises, edema, homeopathy, inflammation, pain, phytotherapy, sport, surgery, trauma

## Abstract

*Arnica* L. genus (Asteraceae) comprises perennial herbs native to the temperate and boreal parts of the northern hemisphere. *Arnica montana* is the main species. It shows different biological activities, such as antioxidant, anti-inflammatory, antibacterial, antifungal, and antitumor effects. The *Arnica* formulations are mainly used for pain management. This systematic review is aimed at summarizing the studies focusing on the use of *Arnica* products on pain and inflammatory signs due to traumatic injuries related to sport and surgical interventions as well as to arthritis and other inflammatory conditions. Both phytotherapeutic and homeopathic formulations are taken into account. This paper only includes manuscripts published in mainstream journals. A literature search from Scopus, Web of Science, and PubMed databases has been carried out using a combination of the keywords “*Arnica*”, “trauma”, “sport”, “injury”, “injuries”, and “pain”. According to the search strategy and inclusion criteria for this study, 42 eligible papers, focusing on both *Arnica* alone and formulations containing a mixture of plant extracts, have been finally selected. This review critically discusses the in vitro, in vivo, and clinical studies dealing with *Arnica* products, reporting both positive and negative outcomes, thus providing perspectives for future research on the plant pharmacological potential.

## 1. Introduction

*Arnica* L. genus belongs to the Asteraceae family and, according to The World Flora Online (WFO) database (https://www.worldfloraonline.org/, accessed on 12 March 2024), it currently comprises 33 known species: *A. acaulis* Britton, Sterns & Poggenb., *A. angustifolia* Vahl, *A. attenuata* (Greene) Maguire, *A. cernua* Howell, *A. chamissonis* Less., *A. cordifolia* Hook., *A. dealbata* (A. Gray) B.G.Baldwin, *A. denudata* Greene, *A. discoidea* Benth., *A. fulgens* Pursh, *A. gracilis* Rydb., *A. griscomii* Fernald, *A. intermedia* Turcz., *A. lanceolata* Nutt., *A. latifolia* Bong., *A. lessingii* Greene, *A. lonchophylla* Greene, *A. longifolia* D.C. Eaton, *A. louiseana* Farr, *A. mallotopus* Makino, *A. mollis* Hook., *A. montana* L., *A. nevadensis* A. Gray, *A. ovata* Greene, *A. parryi* A. Gray, *A. porsildiorum* B. Boivin, *A. rydbergii* Greene, *A. sachalinensis* (Regel) A. Gray, *A. sororia* Greene, *A. spathulata* Greene, *A. unalaschcensis* Less., *A. venosa* H.M. Hall, and *A. viscosa* A. Gray [1].

Plants belonging to this genus are perennial herbs native to the temperate and boreal parts of the northern hemisphere [2].

*Arnica montana* and, to a latter extent, other *Arnica* species have been largely utilized as a popular remedy to treat pain and bruises [3].

*A. montana* is usually referred to as simply “arnica”, and it is also known as “mountain tobacco” or “leopard’s bane” [4]. This species has been traditionally used to treat different ailments, and it has been demonstrated to show antioxidant [5,6], anti-inflammatory [7,8,9], antibacterial [10,11], antifungal [12], and antitumor activity [13,14]. This species is found in Europe and has been introduced into the Tropical Asia region [1].

*Arnica* products are widely used for the management of pain due to a number of conditions, such as musculoskeletal diseases, arthritis, and surgery [15]. Given the very interesting biological activities that characterize *A. montana* species, previous reviews have summarized various aspects of the phytochemical and pharmacological properties of this species. In 2014, Brito and colleagues reviewed the efficacy of the topical administration of *A. montana* in the treatment of pain, swelling, and bruises [3]. The authors highlighted that the efficacy at doses below 10% was not supported by any evidence, and underlined the need for further studies to assess the effectiveness and safety of higher doses. One year later, in 2015, the effects of homeopathic dilutions of *A. montana* on traumatic tissue injury were described by Charlton, who highlighted a number of pieces of conflicting information, including bias/rhetoric for and against the use of this remedy for the treatment of traumatic tissue injuries [16].

Especially in the past years, many papers dealing with the potential use and beneficial properties of *Arnica* have been published in homeopathic journals, and the methodological quality of the investigations described was often poor [17]. Although homeopathic remedies are frequently used by many health professionals, there is an ongoing debate concerning their effectiveness. Currently, no unifying explanation on the mechanisms that could explain how homeopathy works exists [18]. This review, as underlined in the following methodology section, takes into account the manuscripts published in mainstream journals and indexed in the main databases.

This systematic review aims to offer an overview of the in vitro, in vivo, and clinical studies on the use of both phytotherapeutic and homeopathic *Arnica* formulations on pain and inflammatory signs due to traumatic injuries related to sport and surgical interventions as well as to inflammatory conditions including arthritis and other diseases. Positive and negative outcomes are presented and discussed.

## 2. Methodology

This review was performed according to the Preferred Reporting Items for Systematic Reviews and Meta-Analyses (PRISMA) guidelines [19]. Scopus, Web of Science, and PubMed search engines were used. The keyword “*Arnica*” was combined with the keywords “trauma”, “sport”, “injury”, “injuries”, and “pain” by using Boolean operators “and” and “or”, in order to obtain more targeted results.

Moreover, a machine learning-based approach was adopted using the MySLR digital platform [20], a semi-automated tool supporting scientists in performing systematic literature reviews (SLRs), available upon registration at https://myslr.unical.it (accessed on 2 March 2024). This tool allowed us to use a methodological approach that included different steps, namely paper selection, their analysis, and the synthesis of the results.

The first step consisted of the selection discussed above. Overall, 572 papers were retrieved, of which 312 were found in the Scopus database, 136 on the Web of Science, and 124 on the PubMed search engine. In the second step of the bibliographic search, duplicates (211) were removed and the remaining papers (361) were manually screened on the basis of their title and abstract in order to select those papers that fitted the eligibility criteria. The following inclusion and exclusion criteria were adopted: studies published in English and articles whose title and/or abstract referred to the specific use of *Arnica* formulations on traumatic injuries or correlated pain and inflammation were included, while book chapters, previous reviews, retracted articles, and letters to editors were excluded, together with papers not in English.

After this primary screening, 298 manuscripts were manually excluded and 9 full-papers were not retrieved. The remaining 54 full-text records were deeply inspected. Finally, 42 references were included in this review.

**Figure 1:**
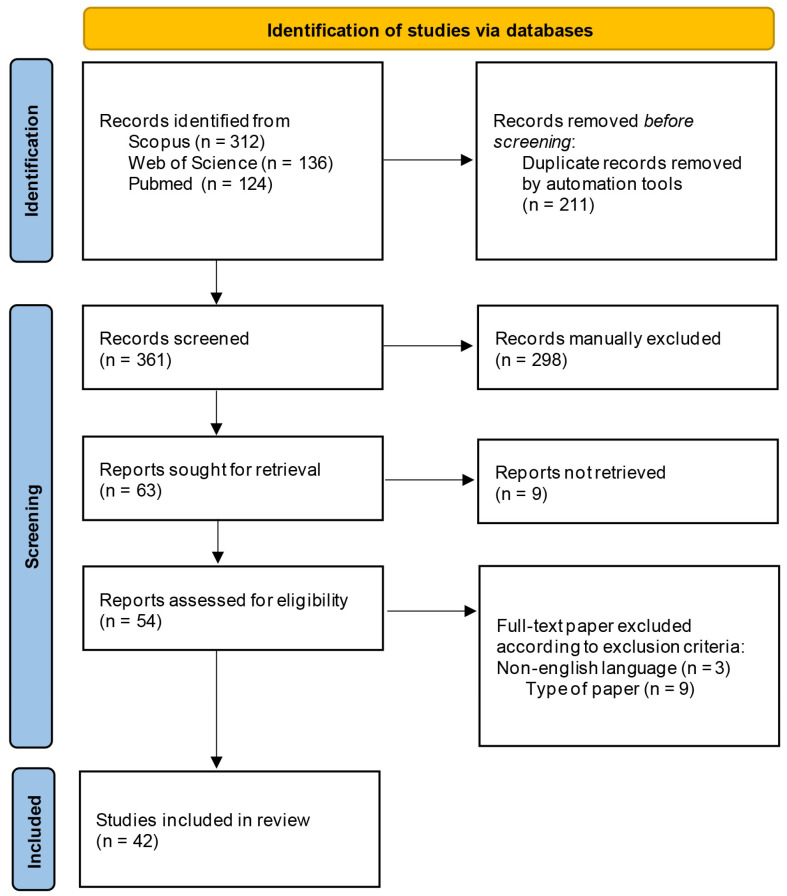
Selection process of the eligible papers based on the PRISMA 2020 flow diagram.

Figure 1 describes the selection process of the bibliographic sources cited in this review.

**Figure 2:**
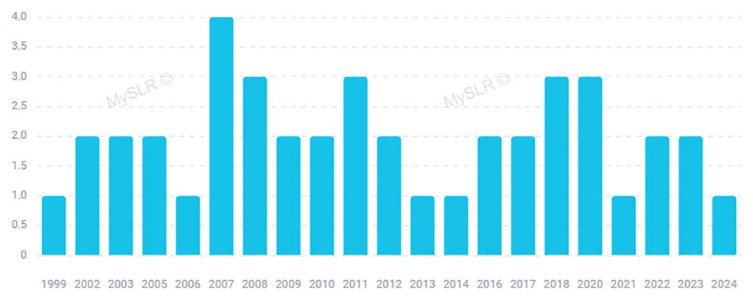
Papers included in this review distributed by year (created with MySLR).

**Figure 3:**
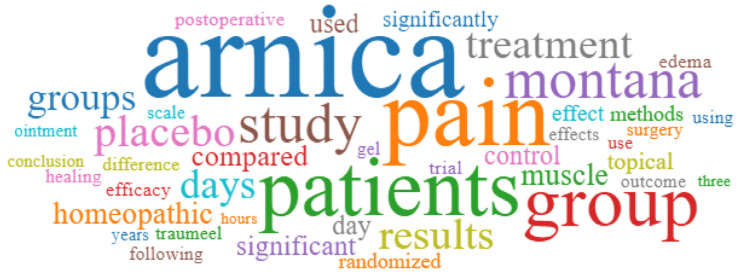
Word cloud highlighting the importance of keywords (Produced by MySLR platform).

As already mentioned, a number of papers focusing on *Arnica* have been published in some homeopathic journals that are not indexed in the main databases [17] and they have not been included in this review. However, starting from 1999 up to the present date, it is possible to observe a constant interest on this topic by mainstream journals, and both in vivo studies and clinical trials have been carried out to investigate the potential health benefits of *Arnica* on traumatic injuries and inflammatory diseases. Figure 2 reports the distribution of the selected papers by years of publication. Figure 3 illustrates the most significant keywords, visually represented through a “word cloud”.

## 3. Commonly Used Routes of Administration and *Arnica montana* Phytochemical Content

*A. montana* is usually administered topically in pharmacological doses, or orally in very diluted doses that do not present side effects as a homeopathic drug [3]. Indeed, when ingested this herbal remedy can induce vascular dilation, blood stasis, and increased capillary permeability [21]. According to the homeopathy’s main principle “like cures like”, a substance in small amounts is able to treat those symptoms that in the healthy individuals are due to higher doses of the same compound [22]. As a homeopathic remedy, *Arnica* dilutions are expressed in Roman numeral units. Dilutions 1:10 are labeled with “X” (or “D”, decimal scale); “C” (centesimal scale) corresponds to dilutions 1:100; and dilutions 1:1000 are denoted as “M” (millesimal scale). The unit “LM” (or “Q”, fifty millesimal scale) refers to dilutions 1:50,000. According to these dosages, arnica 10X and arnica 10C have been diluted 10 times 1:10 and 10 times 1:100, respectively [4,23]. The potency of homeopathic arnica, as well as other homeopathic remedies, is considered to be inversely related to the concentration of the remedy. The more times it has been diluted, the more potent the remedy is thought to be [24].

According to the European Pharmacopoeia, the *A. montana* tincture is obtained from the flowers and should contain 0.04% sesquiterpene lactones expressed as dihydrohelenalin tiglate, and it contains one part of the drug in ten parts of 60% (*v*/*v*) to 70% (*v*/*v*) ethanol. According to European Union, the herbal *A. montana* preparations include flower tincture 1:10 extracted with ethanol 70% *v*/*v* or 1: 5 extracted with ethanol 60% *v*/*v* and liquid extract (1:20) obtained with ethanol 50% *m*/*m* [14].

Sesquiterpene lactones (STLs) of the helenanolide type, i.e., helenalin, 11a,13-dihydohelenalin, and their short-chain carbonic acid esters, are among the most important phytoconstituents identified in *A. montana* species, together with flavonoids, fatty acids, monoterpenes, and sesquiterpenes [14]. STLs are the most important phytochemicals of these species, as they are considered the active principles responsible for the pharmacological importance of *Arnica*. They include different esters of helenalin and 11,13-dihydrohelenalin with low-molecular-weight carboxylic acids, namely acetic, isobutyric, methylbutyric, methacrylic, and tiglic acid. Small amounts of the unesterified parent STLs are also present [25]. Phenolic acids including chlorogenic acid, caffeic acid, and cynarin have been also detected, as well as carotenoids, diterpenes, pyrrolizidine alkaloids, and polyacetylenes. Some coumarins, i.e., umbelliferone and scopoletin, have been identified as well [14,26,27,28,29]. It has been observed that the level of flavonoids and caffeic acid derivatives may also vary depending on the altitude of the growing site [30]. Moreover, the presence of some lignans have been detected by Schmidt and colleagues, who identified these components in *A. montana* and three other *Arnica* species, namely *A. angustifolia*, *A. lonchophylla*, and *A. chamissonis* [31]. The essential oils from the roots and rhizomes of *A. montana* are particularly rich in 2,5-dimethoxy-*p*-cymene and 2,6-diisopropylanisole [32].

## 4. In Vitro and In Vivo Studies Concerning *Arnica montana* L. Formulations

*A. montana* is the most investigated species belonging to the *Arnica* genus. The beneficial effects of both phytotherapeutic and homeopathic formulations of *A. montana* were assessed in different in vitro studies (Table 1).

One of these interesting studies was carried out by Verre and coworkers, who verified the effects of *A. montana* tincture and homeopathic dilutions on inflammation markers and oxidative stress in different human and murine cell culture models [33]. The mother tincture was prepared using 45% ethanol (*v*/*v*) at a ratio of 1/10, and the homeopathic dilutions 1C, 3C, 5C, and 9C were also tested. The potential anti-inflammatory properties were measured evaluating the levels of the inflammatory markers TNFα, IL-6, COX-2, MCP-1, ICAM-1, as well as oxidative stress compounds (ROS). Both mother tincture and 1C dilution significantly reduced TNFα production in inflamed macrophages and IL-6 and MCP-1 in inflamed human microglial cells. COX-2 expression was also significantly decreased in inflamed murine fibroblasts. Moreover, the expression of ICAM-1 was significantly reduced by all the *Arnica* formulations tested in inflamed human endothelial cells compared with the vehicle. As regards the effects on the oxidative stress, a significant consistent effect on ROS production in inflamed murine microglial cells was observed, with 1C dilution being the most effective sample [33].

Jäger and colleagues assessed the potential anti-arthritic activity of the flower head tincture, obtained through the extraction of the fresh plant material with ethanol 70% *v*/*v*. The extract was tested on human and bovine articular chondrocytes [34]. The *Arnica* sample was demonstrated to suppress MMP1 and MMP13 mRNA levels in a concentration-dependent manner in both cell lines. These collagenases are enzymes involved in the pathological destruction of cartilage [37,38]. The effects induced by the *Arnica* sample were supposed to be due to the inhibition of DNA binding of the transcription factors AP-1 and NK-kB [34].

Das and coworkers investigated the ability of *A. montana* to repair the DNA damage. The experiments were performed on *E. coli* exposed to ultraviolet irradiation, and the homeopathic dilution 30C was evaluated. Bacteria were treated with the *Arnica* sample, and exposed to UV light at 25 and 50 J/m^2^. Compared to the control, treated bacteria showed a lesser DNA damage. Moreover, a decrease in ROS generation and an increase in SOD, CAT, and GSH activities were observed following *Arnica* treatment, demonstrating a lesser oxidative stress compared to untreated bacteria. The up-regulation of repair genes was also demonstrated using the reverse transcription polymerase chain reaction method [35].

As the oxidative damage is associated with the pathogenesis of various diseases, including inflammation, arthritis, and also trauma [39,40,41], the evaluation of the antioxidant potential of the *A. montana* sample appears to be useful for the evaluation of its potential health beneficial properties and its anti-inflammatory potential. The antioxidant potential of *A. montana* was reported by Gawlik-Dziki and colleagues, who assessed the in vitro biological properties of ethanolic extracts from *A. montana* flower heads and rhizomes. The extracts demonstrated chelating power ability and lipid peroxidation inhibitory potential. Moreover, an in vitro lipoxygenase inhibitory activity was observed [36].

The biological properties of the species were also assessed through in vivo studies, some of which focused on the potential health benefits of *Arnica* topical formulations (Table 2).

Alfredo and coworkers investigated the effects of a phytotherapeutic formulation of *Arnica* gel containing 200 mg of tincture per g, both associated with phonophoresis and alone [42]. The study was performed on 40 Wistar male rats. The tibialis anterior muscle was surgically lesioned, and animals were divided into four groups receiving, respectively, no treatment; a session of phonophoresis (3 min) daily for 2 days; phonophoresis plus *Arnica* gel with the same scheme of application; and a massage with *Arnica* formulation without phonophoresis. The histological analyses performed on inflamed muscle revealed that the *Arnica* group presented a lesser density of polymorphonuclear cells when compared to the other ones, while no significant differences were found between phonophoresis and phonophoresis-plus-*Arnica* groups. The massage with *Arnica* gel was reported to have a significant anti-inflammatory effect on acute muscle lesion in topic use, while *A. montana* phonophoresis proved to be ineffective.

Sharma and colleagues investigated the in vivo anti-arthritic potential of *A. montana*. The flower methanolic extract was tested on collagen-induced arthritis in rats. Animals were treated with a dosage of 75 mg/kg body weight administered orally for 20 days, and the effects of the treatment were compared to those induced by dexamethasone (1 mg/kg) and the vehicle. The *Arnica* extract was demonstrated to induce a lower expression of tumor necrosis factor-α, interleukins IL-1β, IL-6, and IL-12, nitric oxide, and of the titer of the anti-type II collagen antibody compared with untreated rats, also showing antioxidant potential [43].

Furthermore, *A. montana* was also explored for its the beneficial properties in the treatment of skin disorders related to inflammatory conditions. Da Silva Prade and colleagues investigated the anti-inflammatory effects on a UVB radiation-induced skin–burn model [8]. Male Swiss mice were first exposed to UVB radiation and then treated with an ointment containing *A. montana* (250 mg/g) on the ear. The positive control group received dexamethasone. Both edema and the inflammatory response were measured 16 h after the treatment, together with the oxidative stress. The authors reported that *A. montana* treatment reduced the UVB-induced inflammation, as a reduction in ear edema was observed, together with an inhibition of the myeloperoxidase activation. Moreover, the levels of proinflammatory cytokines, such as IL-1β, IL-6, TNF-α, and IFN-γ, were reduced, and the UVB-induced oxidative damage was ameliorated.

## 5. Clinical Trials Evaluating the Efficacy of *Arnica montana* L. Formulations

In addition to the preclinical studies reported in the section above, several clinical trials focusing on the use of phytotherapeutic or homeopathic *A. montana* formulations have been performed. Table 3 summarizes these studies, reporting the kind of extract/sample, the study design, the treatment, and the obtained results of each study, classifying them according to the type of use and clinical application tested.

### 5.1. Muscle Pain and Bruising

Many therapeutic interventions have been tested to alleviate muscle soreness. One of the difficulties for researchers is to find a large pool of participants with similar muscle injuries. The delayed onset muscle soreness (DOMS), a common result of muscular overexertion in unusual exercisers, is useful for researchers to carry out a systematic investigation, as DOMS symptoms are similar to many athletic injuries [24]. A phytotherapeutic formulation of *Arnica* was tested for its effects on muscle pain and performance. Pumpa and colleagues reported a double-blind, randomized placebo-controlled trial involving 20 well-trained males, and focused on the effectiveness of an *A. montana* gel containing tincture equiv. to dry flower herb 10 mg per g. The formulation was tested for its ability to reduce pain and improve performance in well-trained males with DOMS. The gel was topically applied to the skin on the quadriceps and gastrocnemius muscles immediately after a downhill running protocol designed to induce DOMS, and applied every 4 waking hours. The formulation was demonstrated to provide pain relief 72 h post-exercise, even if it did not affect any markers of muscle damage or inflammation [44].

Leu and colleagues described a rater-blinded randomized controlled trial on the ability of a topical 20% *Arnica* formulation to enhance the resolution of laser-induced bruising. In this study, involving healthy volunteers aged between 21 and 65, four bruises of 7 mm diameter each were created on the bilateral upper inner arms, using a 595 nm pulsed-dye laser. The patients were divided into four groups, each randomly receiving 5% vitamin K, 1% vitamin K, 0.3% retinol, 20% *Arnica*, or white petrolatum. The improvement due to the treatment with 20% *Arnica* was greater than the placebo and the mixture of 1% vitamin K and 0.3% retinol, even if it was not higher than that induced by 5% vitamin K cream [45].

### 5.2. Postoperative Pain, Edema, and Wound Healing

Different therapies have been taken into account to improve pain control after surgical procedures, including complementary therapies. Among these, the role of homeopathic *Arnica* has been investigated, for example, to control pain following ambulatory knee surgery, as after this kind of procedure patients are notoriously encouraged to move the knee [57]. The success of the intervention is closely linked also to the control of postoperative pain, usually based on the use of systemic or intra-articular application of non-steroidal anti-inflammatory drugs (NSAIDs) or opiates [46].

Brinkhaus and colleagues described three randomized, double-blind trials designed to assess the effectiveness of homeopathic *Arnica* in decreasing swelling and pain following knee surgery (Table 3) [46]. The authors took into account three different conditions: knee arthroscopy, artificial knee joint implantation, and cruciate ligament reconstruction. Participants enrolled in the trials (227, 35, and 57, respectively) were patients of both genders whose age was between 18 and 75 years, and who were suffering from knee diseases needing surgery. These studies aimed to verify the efficacy of arnica 30X administered orally using sucrose globules. Five globules were given to patients three times a day from the day of surgery to the end of the follow-up examination. The authors reported a significantly reduced postoperative swelling in patients receiving homeopathic arnica compared to those receiving the placebo in the trial concerning the cruciate ligament reconstruction, while just a trend towards a lesser swelling was observed in the other two examined conditions.

An interesting study was described by Jeffrey and Belcher [17], who took into account the combined administration of both homeopathic and phytotherapeutic *Arnica* formulations to relieve pain following carpal tunnel release surgery. The treatment was based on the use of the homeopathic arnica D6 tablet and an ointment containing the 10% *w*/*v* of *A. montana* ethanolic extract. Forty patients were entered into the clinical trial and divided into an *Arnica* and a placebo group. The administration of *Arnica* tablets started from the day of surgery for two weeks, and the ointment was administered through a gentle massage three times daily during the same period. A significant reduction in pain was observed after 2 weeks of treatment, while any difference in grip strength and wrist circumference was found between the two groups.

Furthermore, the potential analgesic effects of homeopathic arnica were also verified after tonsillectomy. A randomized, double-blind, placebo-controlled trial, involving 190 patients aged over 18 years and undergoing tonsillectomy was reported by Robertson and colleagues. Patients were divided into two groups: receiving arnica 30 CH or placebo tablets. Two tablets six times on the first postoperative day and then two twice a day for the next seven days were administered. A small but statistically significant decrease in pain scores was observed compared to the placebo [47].

Macedo and colleagues evaluated the efficacy of homeopathic arnica 6 CH in 32 patients who underwent the extraction of impacted third molars. In the described crossover and double-blind study, the authors assessed the impact on edema, pain, and also mouth opening compared to the placebo. The patients received five drops sublingually three times a day before surgery, followed by five drops two times a day at the day of surgery, and three times daily the first day after surgery. Even if any effect was observed on pain and mouth opening, the treatment significantly reduced edema compared to the control group [48].

The homeopathic tablets arnica 30X were tested for their efficacy on the postsurgical sequels following extraction of impacted mandibular third molars as well [49]. Mawardi and coworkers described a case–control pilot study involving 23 patients, which were divided into a treatment and a control group. They received four tablets 1 h before the procedure and four tablets four times per day starting 1 h after the procedure and for the three following days. The treatment induced a significant reduction in pain, bleeding, bruising, and edema, and a decrease in maximum mouth opening compared to the control group.

The homeopathic formulation 200C was also tested for its effects on post-extraction pain management. In a triple-blind randomized controlled trial, 44 children aged between 8 and 12 years requiring two clinical sessions of tooth extraction in two different quadrants of the oral cavity were selected. In this crossover trial, all the children received both arnica and ibuprofen with a washout of 10 days. Authors reported that no differences were observed between arnica and ibuprofen in the post-extraction pain management, suggesting that arnica may be considered as an alternative to ibuprofen in managing post-extraction pain in 8–12-year-old children [21].

The efficacy of homeopathic arnica capsules was tested for its beneficial effects in reducing edema associated with rhinoplasty. Forty-eight patients were randomly treated with arnica or dexamethasone three times a day for 4 days. Even if arnica did not provide any benefit with regard to the extent of ecchymosis, the study suggested that it was effective in reducing edema during the early postoperative period following rhinoplasty [50].

Karow and colleagues focused on the wound healing properties of Arnica after surgery, rather than the decrease in pain. In a randomized double-blinded, parallel-group study involving 88 patients, the authors investigated the efficacy of arnica 4D pills administration in healing the wounds due to hallux valgus surgery [51]. The efficacy of arnica was compared to those of diclofenac sodium (50 mg per os, three times daily). Both this drug and arnica 4D (10 pills three times per day) were administered to patients undergoing surgery for hallux valgus for 4 days following the intervention. Both treatments were equivalent as for wound irritation and patient mobility. On the contrary, arnica showed a lesser analgesic activity but was better tolerated than diclofenac.

Mendes and coworkers evaluated the beneficial effects of *Arnica* on the cicatrization process of aphtha, the small and painful ulcer on human oral mucosa [52]. Thirty-one patients utilized an ointment containing *Arnica* tincture. The formulation was topically applied three times daily. The medication demonstrated anti-inflammatory activity and beneficial effects on the healing process of lesions in oral mucosa.

Interestingly, Nejadbagheri and colleagues evaluated the use of *A. montana* in reducing the pain associated with arteriovenous fistula puncture in patients receiving hemodialysis. In a double-blind single-group randomized clinical trial, patients received an arterial and a venous fistula puncture, randomly allocated to the experiment and the placebo. Before needle insertion, the experiment and the placebo sites were treated for 10 min with a cream containing 5 mg of *A. montana* extract per 100 mg or vitamin A and D ointment, respectively. The treatment was effective in reducing pain [53].

### 5.3. Trauma-Induced Pain

Lower back pain is one of the most common health problems worldwide, and it can be due to different factors, such as overuse, muscle strain, trauma, or injuries to the ligaments, muscles, and discs that support the spine [58]. The homoeopathic arnica has been tested in conjunction with physiotherapy to treat traumatic low backache by Alekar and colleagues, who recruited 30 volunteers whose lower back pain was related to a trauma, while all other causes of backache were excluded from the trial. The patients were of both sexes between 20 and 40 years of age (Table 3) [54]. The subjects were divided into three groups: the group A received a dose of arnica 200 twice a day for 15 days, the group B received constitutional medicine (with *Bryonia alba* Linn., *Rhus toxicodendron* Linn., and *Nux Vomica* Linn. being administered on a constitutional basis), and group C received the placebo. Each of the three groups also received a daily session of physiotherapy. Significant improvement in pain scores was observed, with a more pronounced effect in group B. The authors suggested that an individualized approach was more effective in the management of patients. After 15 days of treatment, an assessment was carried out and the repetition of the dose was decided accordingly.

### 5.4. Osteoarthritis

*Arnica* has been used for centuries in traditional medicine as a remedy for traumatic injuries and inflammatory conditions of the locomotor system [59].

Knuesel and coworkers reported the results of a 6-week multicenter trial focused on the safety and efficacy of an *A. montana* gel formulation and involving 79 patients with mild-to-moderate osteoarthritis of the knee (Table 3). Patients were asked to apply a thin layer of *A. montana* gel to the affected knee two times per day. An amount of 100 g of gel contained 50 g of *Arnica* fresh plant tincture (drug-to-extract ratio 1:20 in 50% ethanol). The study demonstrated that the topical application of the *Arnica* gel was effective, being at the same time safe and well tolerated, with the exception of an allergic reaction that occurred in one of the patients [55].

Widrig and colleagues tested the efficacy of a phytotherapeutic *Arnica* gel formulation (50 g *A. montana* tincture/100 g, with a drug-to-extract ratio of the tincture 1:20) on osteoarthritis of interphalangeal joints the hand. The performed randomized, double-blind study involved 204 patients, randomly treated with ibuprofen or *Arnica* gel. Patients were asked to apply the formulation over the affected joints thrice daily for 3 weeks, and were asked not to wash their hands for one hour after application. At the end of the treatments, no differences between the two groups in pain and hand function improvements were observed. Adverse events were reported by six patients using ibuprofen and by five patients treated with *Arnica*. These results confirmed that arnica gel is not inferior to ibuprofen on the symptoms of osteoarthritis of the hand [56].

## 6. Case Reports

Some case reports focusing on the pharmacological properties of arnica homeopathic formulations have been also reported (Table 4).

Barkey and colleagues described the use of a homeopathic arnica patch (3X) for its anti-inflammatory effects in a 55-year-old female patient with cellulitis-derived pain and numbness in the hand. Arnica patches were applied on the painful areas of the hand at night and a decrease in pain symptoms was reported after three days [60].

Another case report dealt with a phytotherapeutic formulation of *A. montana*. Jackson and Tummon Simmons assessed the beneficial outcomes of the topical application of a 200 mg/mL *A. montana* flowering tops formulation in olive oil (1:5 herb-to-olive oil ratio) in an 82-year-old Caucasian female suffering from severe shoulder deterioration due to osteoarthritis. During the first five weeks, the treatment consisted of the topical application of *Arnica* oil with a massage for about five minutes followed by acupuncture. On the sixth week, therapeutic ultrasound was also introduced. Some positive outcomes were reported, such as a decreased pain score just after the first week of treatment, which was then further reduced following the introduction of therapeutic ultrasound. Moreover, a decrease in medication usage and an improved functionality were observed [61].

## 7. Clinical Studies on Formulations Containing *Arnica* and Other Plant Species

*Arnica* is often used as one of the ingredients of formulations also containing other plant extracts aimed at treating trauma and inflammatory conditions.

Bartolomei and colleagues verified the effectiveness of a mud containing both 3% *A. montana* essential oil (EO) and 5% menthol to provide pain relief after a high-volume resistance training session [62]. A randomized counterbalanced crossover study was conducted on 10 resistance-trained men that were asked to perform a high-volume resistance workout for lower body squats and leg extensions. The volunteers were aged between 18 and 35, with a minimum of 2 years of resistance training experience. Additional mud or placebos were topically applied above the quadriceps muscle of both legs 3, 19, 27, and 45 h after the workout, and muscle performance, morphology, and soreness were measured before and after the exercise. The observed results allowed us to conclude that muscle morphology was not influenced by mud packs, and that mud with *A. montana* could be useful to enhance the recovery rate of strength and to reduce muscle soreness after high-volume exercise (Table 5).

Zanella and colleagues evaluated the effectiveness of Arnica Comp. -Heel^®^ (“Arnica compositum”) ointment in the treatment of symptomatic calcific periarthritis of the shoulder [63]. This homeopathic combined ointment contains a number of plants, namely arnica (*A. montana* L.), calendula (*Calendula officinalis* L.), chamomile (*Matricaria recutita* L.), comfrey (*Symphytum officinale* L.), milfoil (*Achillea millefolium* L.), deadly nightshade (*Atropa belladonna* L.), monkshood (*Aconitum napellus* L.), daisy (*Bellis perennis* L.), St John’s wort (*Hypericum perforatum* L.), narrow-leaved cone flower (*Echinacea angustifolia* DC.), purple cone flower (*Echinacea purpurea* L.), witch hazel (*Hamamelis virginiana* L.), together with mercurico-amidonitrate and calcium sulphide. A pilot study was described, in which 41 patients with calcific periarthritis of the shoulder were treated with a combination of “Arnica compositum*”* ointment (AC) + Acidum nitricum (AN) + Hekla lava (HL) or with *Arnica compositum* ointment alone. Patients were asked to apply the formulations on the shoulder and then to cover the shoulder with a bandage, in order to prevent the immediate removal of the material by rubbing on clothing. After a 3-day therapy, the reduction in pain as well as the improvement of shoulder motion were superior in the group treated with AC + AN + HL ointment mixture compared to the group receiving AC ointment alone.

Differently from the study by Macedo [48], who tested a homeopathic dilution containing just *A. montana*, De Souza and colleagues evaluated the homeopathic preparation Traumeel*^®^* S in the control of postoperative outcomes (pain, edema, and trismus) associated with the surgical removal of mandibular third molar teeth [64]. The remedy Traumeel^®^ (Biologische Heilmittel Heel GmbH, Baden-Baden, Germany), has also been marketed in Italy under the name *Arnica compositum* (Arnica Comp. -Heel*^®^*) [74], so it is the same formulation of 12 botanical and 2 mineral substances already described before (*Achillea millefolium*, *Aconitum napellus*, *A. montana*, *Atropa belladonna*, *Bellis perennis*, *Calendula officinalis*, *Matricaria recutita*, *Echinacea angustifolia*, *Echinacea purpurea*, *Hamamelis virginiana*, *Hypericum perforatum*, *Symphytum officinale*, mercurico-amidonitrate, and calcium sulphide) [64,75]. In the preliminary randomized triple-blind clinical trial described by de Souza, 17 patients received Traumeel*^®^*S or dexamethasone preoperatively by injection into the masseter muscle. Pain, edema, and trismus (a condition of restricted opening of the mouth) levels were observed after 24 h, 72 h, and 7 days. The results obtained for the treatment were not different from those of dexamethasone at all postoperative evaluations, allowing us to conclude that Traumeel^®^ S might be a good alternative approach to dexamethasone for controlling pain, edema, and trismus after third molar removal [64].

In a pilot clinical trial involving 30 patients, Singer and colleagues tested the efficacy of the same formulation on pain following elective *Hallux valgus* surgery [65]. A first group received an injection of Traumeel^®^ S (2.2 mL) into the operative incision upon conclusion of surgery, while a second one received both injection and then tablets orally three times a day for 13 days or until pain was negligible. Both single-injection and injection-plus-oral-treatment groups were effective in lowering pain as compared to the control group, suggesting that the formulation was effective in minimizing postoperative pain following repair of *H. valgus*.

In another study, *A. montana* was combined with *Bellis perennis* in a homeopathic formulation tested for the reduction in seroma following mastectomy and immediate breast reconstruction. The medication was based on the combination of *A. montana* C30 and *Bellis perennis* C30. This randomized, double-blind, placebo-controlled trial involved 55 patients, who were randomly assigned to the treatment with homeopathic medication or the placebo, administered as spherical saccharose globules. Obtained results demonstrated that the formulation was able to reduce seroma formation and also to decrease opioid intake following mastectomy and reconstruction [66].

Traumeel^®^ S was also investigated for its effectiveness in the treatment of tendinopathies of varying etiology, taking into account both pain- and motility-related variables. Schneider and coworkers reported a nonrandomized observational study involving 357 patients with tendinopathies based on an excessive tendon load rather than inflammation. Patients were treated with Traumeel^®^ S ointment or diclofenac 1% gel for a maximum of 28 days. The study demonstrated that arnica homeopathic formulation was not inferior to diclofenac therapy as regards both pain and motility [67].

Janczewska and colleagues tested the analgesic effectiveness of Traumeel*^®^* S ointment in patients with gonarthrosis. The topical treatment was used in combination with magnetic field LED therapy [68]. Magnetic field LED therapy is a physical therapy modality consisting in the combined effects of extremely low-frequency electromagnetic fields and high-power light emitted by light-emitting diodes (LEDs) [76]. Ninety patients with knee osteoarthritis were divided into three groups, and treated with magnetic stimulation plus LED therapy, Traumeel^®^ S ointment, and their combination, respectively. The combined therapy gave positive results in terms of pain reduction, as well as the other two groups, even if the strongest analgesic factor seems to be magnetic and LED therapies [68].

Phytoterapeutic *Arnica* was also investigated for its effects on ankle distortion. A spray formulation combining *Arnica* tincture and hydroxyethyl salicylate was tested on 570 patients with acute ankle joint distortion in a randomized double-blind study [69]. Patients were divided into three groups receiving the combined formulation, *Arnica*, and hydroxyethyl salicylate alone, respectively. The treatment was applied four-to-five times daily for 10 days. A significant pain improvement after the combination spray administration was observed compared to *Arnica* and hydroxyethyl salicylate groups.

The same combined formulation, a combination spray of *Arnica* tincture and hydroxyethyl salicylate, was also tested for its analgesic properties and the potential use in sports injuries and diseases of the locomotor apparatus [70]. In this randomized, single-blind trial on 40 healthy volunteers, each subject received both the drug and placebo, filled into white 50 mL plastic bottles with a spray top. The spray was used on a defined skin area, the inner side of the forearm. The combination preparation was more effective than both the placebo and the individual active components.

Madisetti and colleagues tested a topical wound powder containing *A. montana* 0.01% *v*/*w* together with other plant species, including *Calendula officinalis* L., *Mentha arvensis*, and *Santalum album*. The pilot study demonstrated the feasibility, acceptability, and tolerability of a wound care powder for hospice patients with chronic wounds at the end of their life [71]. The effects of this formulation on pain and exudate in 50 patients with ulcers and vascular wounds receiving palliative care at the end of their life was also described in another study, reporting a significant reduction in both pain and exudate [72].

The potential beneficial effects of *Arnica* containing formulations were also taken into account for the treatment of burns. Huber and coworkers reported a case study in which two individuals used the formulation Combudoron *^®^* (Weleda AG Schwäbisch Gmünd, Germany), containing the ethanolic extracts of *Urtica urens* and *A. montana*, for the treatment of laser-induced burns. Four experimental-grade burns (1 cm^2^) were induced with a laser and the lesions were treated for 15 days with Combudoron gel, Combudoron liquid, placebo gel, or placebo liquid in each of the subjects. The treatment seemed to have positive effects on burns [73].

## 8. Other *Arnica* Species

Besides *A. montana*, another *Arnica* species has been also evaluated for its potential health benefits in inflammatory diseases and trauma. The tinctures from different parts of *Arnica chamissonis* Less. were demonstrated to exert an in vitro radical scavenging activity, chelating power ability, and lipid peroxidation inhibitory potential. Moreover, this species showed xanthine oxidase inhibitory activity. This study, by Gawlik-Dziki and colleagues, demonstrated that this species could be also useful in the prevention and treatment of free radical-dependent diseases (Table 6) [36].

## 9. In Vivo Studies and Clinical Trials with Negative Outcomes

As underlined by Fanelli [77] and by Matosin and coworkers [78], the discussion of negative outcomes is very important in science, as results that do not confirm the expected effect, being both not statistically significant and in contradiction with the hypothesis, are important for science as the positive ones, as they allow a collective self-correcting process. Negative results are valuable data from the scientific literature as they allow a critical evaluation and validation of our current thinking [78].

In addition to all the positive results previously discussed, some of the studies dealing with the beneficial properties of *Arnica* also reported negative outcomes (Table 7).

Adkison and coworkers investigated the effectiveness of a commercially available *Arnica* homeopathic cream formulation 1X HPUS-7% [4]. HPUS is an acronym that stands for Homeopathic Pharmacopeia of the United States, and indicates that the remedy meets defined criteria, as stated in the Homeopathic Pharmacopeia, such as strength, quality, purity, packaging, or label requirements [22]. The study by Adkison et al. involved 55 male and female volunteers between the age of 18 and 65 years, with both legs functioning without acute or chronic pain. In this randomized, placebo-controlled, and double-blind study design, each participant received two containers of cream produced by the same manufacturer, marked as “left” and “right”. One of the tubes held a commercially available arnica cream formulation, and the other one held the vehicle–placebo cream without the active ingredient. The subjects were asked to apply the formulations to the legs immediately after the exercise, and also at 24 and 48 h post-exercise, and to rate the pain in each leg at the baseline, and after 24, 48, and 72 h. The average amount of cream applied by each subject was found to be 5 g. A negative outcome was observed in this study, since arnica cream was found to increase leg pain 24 h after calf exercises rather than decreasing it. Any significant difference was instead observed among *Arnica* and placebo after 48 and 72 h [4].

The absence of effectiveness was reported also in some studies concerning the use of *Arnica* formulations on postoperative pain and bruising. An in vivo study on the analgesic properties of homeopathic arnica was performed on the control of postoperative pain in cats subjected to a hysterectomy with bilateral salpingo-oophorectomy. The analgesic effects of different formulations and routes of administrations were tested, namely arnica 30X per subcutaneous route (SC), and per oral transmucosal route (OT), and arnica 6X per OT. Their efficacy was compared to those of morphine and ketoprofen. The obtained results demonstrated that arnica showed less efficacy than ketoprofen and morphine. Furthermore, no significant difference was detected either between the different formulations or between the routes of administration [79].

Differently from the randomized, double-blind study from Jeffrey and Belcher, who observed a significant pain reduction in patients undergoing carpal tunnel release surgery [17], negative outcomes related to the use of *Arnica* were described by Stevinson and colleagues [80]. These authors reported a double-blind, placebo-controlled, randomized trial involving 64 adults undergoing surgery for carpal tunnel syndrome. Patients were treated with homeopathic arnica 30CH or 6CH or placebo tablets, which were administered three times daily for seven days preoperatively and fourteen days postoperatively. Patients were asked not to swallow the tablets, in order to have an oral transmucosal route of administration. No significant effects were observed in reducing postoperative pain, bruising, and swelling compared to the placebo.

Homeopathic *Arnica* was also a component of a formulation tested for its ability to reduce analgesic morphine consumption following knee ligament reconstruction. This phase III randomized placebo-controlled study was reported by Paris and coworkers and involved 158 patients undergoing knee ligament reconstruction. The complex tested contained *A. montana* 5 CH, *Bryonia alba* 5 CH, *Hypericum perforatum* 5 CH, and *Ruta graveolens* 3 DH. The activity of the tested formulation was not higher than the placebo [81].

Lauche and colleagues reported a randomized controlled trial focused on the effectiveness of a cupping massage using *A. montana* oil compared to progressive muscle relaxation in patients with chronic non-specific neck pain [82]. Sixty-one patients were randomly divided into two groups, treated for 12 weeks with a partner-delivered home-based cupping massage with *Arnica* oil, or underwent progressive muscle relaxation for the same period. Even if both groups showed significantly less pain compared to the baseline, any significant difference was detected among the two groups, with the cupping massage being no more effective than progressive muscle relaxation in reducing chronic non-specific neck pain.

Differently from Pumpa, who successfully tested the effects of an *Arnica* phytotherapeutic formulation on DOMS [44], Tuten and colleagues did not obtain positive outcomes when testing the sublingual administration of homeopathic arnica 6X pellets [24]. Twenty-three university students aged between 18 and 25 were instructed to take three pellets sublingually before following a bench-stepping protocol in a double-blind randomized study. The obtained results allowed us to conclude that arnica at 6X potency was not an efficacious agent for moderating DOMS.

## 10. *Arnica* Absorption and Side Effects

*Arnica* preparations are generally considered safe, both if used topically on intact skin and orally in homeopathic doses. However, some side effects, such as a rash, dry skin, and itching, have been reported [4].

Moreover, commercialized *Arnica* preparations are often not standardized, and for this reason it is not possible to accurately recommend a dose [23].

As regards the use of *Arnica* in homeopathic preparations, the homeopathic remedies are subject to regulatory control in several countries, such as the European Community, USA, Canada, and Australia. However, despite a long experience and the common perception of safe use, comprehensive toxicological data for the most homeopathic remedies are often scarce or even lacking. Moreover, homeopathic medicines can contain a multitude of different homeopathic ingredients, including raw material of botanical origin, environmental materials such as contaminants, food additives, metals, and even starting materials of biological origin such as micro-organism preparations. The abundance and diversity of homeopathic ingredients make an appropriate safety evaluation difficult [22].

Some sensitizing effects have been related to the use of *A. montana* and related species, above all induced by self-treatment with *Arnica* tincture. These skin reactions have been demonstrated to be due to the sesquiterpene lactones helenalin, its acetate, and methacrylate [83,84].

The absence of toxic effects was instead reported in an in vivo study by Jürgens and coworkers, who tested the dermal application of *Arnica* tincture in rats for the potential treatment of cutaneous leishmaniasis [85].

To assess the dermal absorption of the *Arnica* tincture and its bioactive constituents, STLs, dermal absorption experiments on porcine and human skin (“ex vivo”) in diffusion cells under near-physiological conditions were also performed. In this research, Jürgens and colleagues estimated the amounts of STLs on the skin surfaces, in skin extracts, and in the receptor fluids by UHPLC-HRMS. Within 48 h, a maximum of about 8% and 36% of STLs permeated through porcine and human skin. A significant portion of sesquiterpene lactones did not permeate and could not be extracted from the skin because of an irreversible bounding to skin proteins [86].

## 11. Future Perspectives

An important aspect about the pharmacological potential of *A. montana* has been recently raised by Schmidt, who underlined some important differences among *A. montana* populations from different geographical areas. It is well known that STLs of the helenanolide type, including a variety of esters of helenalin and 11,13-dihydrohelenalin with low-molecular-weight carboxylic acids, are the main active principles responsible for the *Arnica* biological potential. However, a great difference has been observed between *A. montana* flowers from plants growing in Central or Eastern Europe, containing above all helenalin esters, and those originating from the Iberic peninsula, which contain almost exclusively 11,13-dihydrohelenalin derivatives. The presence of two *A. montana* subspecies have also been proposed: subsp. *montana*, mainly occurring in Central and Eastern Europe, and subsp. *atlantica*, present in the Iberian Peninsula [25]. From the point of view of the systematic botany, this difference has not been cited in the recognised database, The World Flora Online (WFO) (http://www.worldfloraonline.org/, accessed on 27 August 2024) [1], in which only *Arnica montana* subsp. montana is currently mentioned as a subspecies of *A. montana*, while *Arnica montana* subsp. *atlantica* A.Bolòs is mentioned as a subspecies of *Arnica chamissonis* Less. However, the presence of two chemotypes is reported to be evident, and the need to compare their pharmacological activity through in vitro, in vivo, and even clinical studies has been proposed in order to assess their efficacy and safety [25].

Moreover, another aspect to be taken into account should be the utilized extraction technique. It is well known that the qualitative and quantitative profile of the phytochemical composition of an extract from plant material depends on the selection of the proper extraction method [87]. Nowadays, response surface methodology (RSM) in extraction processes represents a useful tool for the optimization of the procedures in analytical chemistry and in phytochemistry [88,89]. Garcia-Oliveira and colleagues, for example, investigated how to improve the extraction of the phenolic compound from *A. montana* flowers through multivariate optimization of heat- and ultrasound-assisted methods, suggesting a potential industrial application of *A. montana* flower extracts with an improved resource utilization compared to conventional extraction methods [90]. The same kind of study should be helpfully applied to the extraction of STLs and other bioactive compounds.

## 12. Concluding Remarks

In their review published in 2015 and including eight clinical trials, Ernst and Pittler concluded that homeopathic *Arnica* could be not considered more effective than a placebo [91]. Some years ago (2016), Iannitti and colleagues focused on the use of *Arnica* on just postsurgical pain and inflammation. These authors evidenced that overall *A. montana* could be considered an alternative to non-steroidal anti-inflammatory drugs [92].

In the present review, following a selection process of eligible papers performed according to the PRISMA guidelines, 42 studies dealing with the effects on pain and inflammatory signs due to both traumatic injuries and inflammatory conditions of *Arnica* phytotherapeutic and homeopathic formulations have been discussed. Some of these works have been reported as in vitro and in vivo studies, while most of them were related to clinical trials.

Even if some studies report negative outcomes, a relevant number of evidences support the use of *Arnica* formulations for the treatment of pain, bruises, and swelling that occur after traumatic injuries related to sport and surgical interventions as well as with arthritis and other inflammatory conditions. These positive results refer to *Arnica* as both a single remedy and as a combination with other active principles and remedies.

## Figures and Tables

**Table 1:** In vitro studies concerning the effectiveness of *Arnica montana* L. phytotherapeutic and homeopathic formulations.

Use	Form.	Sample/ Extract	In Vitro Model	Treatment	Results	Ref.
Inflammation	P, H	*A. montana* tincture (45% ethanol at a ratio of 1/10); 1C, 3C, 5C, and 9C homeopathic dilutions	Human and murine cell culture models	Cells treated with sample	Mother tincture and 1C dilution of *A. montana* showed anti-inflammatory effects reducing pro-inflammatory cytokines, adhesion molecules, and ROS in cell models	[33]
Arthritis	P	*A. montana* tincture (flower heads extracted with ethanol 70% *v*/*v*)	Bovine and human articular chondrocytes	Cells treated with sample	Suppression of collagenases MMP1 and MMP13 mRNA levels	[34]
DNA damage	H	*A. montana* 30C	Bacteria (*E. coli*)	UV-exposed bacteria treated with UV light at 25 and 50 J/m^2^	Reduced DNA damage and oxidative stress. Repair genes’ up-regulation	[35]
Oxidative stress	P	*A. montana* ethanolic extract	In vitro assays	Treatments with flower heads and rhizome extracts	Chelating power ability, lipid peroxidation inhibitory potential, lipoxygenase inhibitory activity	[36]

Form.: formulation; H: homeopathic; P: phytotherapeutic; Ref.: reference.

**Table 2:** In vivo studies on *Arnica montana* L. phytotherapeutic and homeopathic formulations effectiveness.

Use	Form.	Sample/ Extract	Adm.	Animal Model	Treated Condition/Disease	Treatment	Results	Ref.
Inflammatory muscle lesions	P	*Arnica* gel (200 mg of tincture per g) with and without phonophoresis	T	40 Wistar male rats	Tibialis anterior muscle was surgically lesioned	(1) *Arnica* gel + phonophoresis for 3 min once a day, for 3 days; (2) massage with *Arnica* gel for 3 min once a day, for 3 days	The massage with *Arnica* gel proved to be an effective anti-inflammatory on acute muscle lesion in topic use	[42]
Arthritis	P	*A. montana* flower methanolic extract	O	Female Wistar rats	Collagen-induced arthritis	Rats received a dosage of 75 mg/kg body weight administered orally for 20 days	Lower expression of TNFα, IL-1β, IL-6, IL-12, NO, and anti-type II collagen antibody titer	[43]
Skin burns	P	*A. montana* tincture (250 mg/g)	T	25 male Swiss mice	UVB radiation-induced skin–burn model	After UVB radiation exposure, mice were treated with a topical application of ointment (250 mg/g) in the ear	Reduced inflammatory response with decrease in ear edema and proinflammatory cytokine levels; ameliorated UVB-induced oxidative damage	[8]

Form.: formulation; P: phytotherapeutic; Adm.: route of administration; O: oral; T: topical; Ref.: reference.

**Table 3:** Clinical trials concerning the effectiveness of *Arnica montana* L. phytotherapeutic and homeopathic formulations.

Use	Form.	Sample/ Extract	Adm.	Treated Condition/ Disease	Study Design	Part.	Treatment	Results	Ref.
Muscle pain and bruising	P	*Arnica* gel (tincture equiv. to dry flower herb 10 mg per g)	T	Delayed onset muscle soreness	Double-blind, randomized placebo-controlled trial	20 well-trained males	*Arnica* was applied to the skin superficial to the quadriceps and gastrocnemius muscles immediately after a downhill running protocol and reapplied every 4 waking hours	Topical *Arnica* provided pain relief 3 days post-eccentric exercise	[44]
	P	20% *Arnica* ointment	T	Four bruises of 7 mm diameter each created on the bilateral upper inner arms, using a 595 nm pulsed-dye laser	Rater-blinded randomized controlled trial	Healthy volunteers aged between 21 and 65	*Arnica* ointment was compared to two vitamin K formulations and the placebo on the resolution of skin bruising	The improvement associated with 20% *Arnica* was greater than the placebo, the mixture of 1% vitamin K, and 0.3% retinol, but not greater than 5% vitamin K cream	[45]
Postoperative swelling and pain	H	Arnica 30X	O	Swelling and pain following knee arthroscopy	Randomized, double-blind	227 patients	Arnica 30X administered orally with sucrose globules. Five globules three times a day (from 2 h before surgery to the last follow-up examination)	Trend towards less postoperative swelling	[46]
	H	Arnica 30X	O	Postoperative swelling and pain after artificial knee joint implantation	Randomized, double-blind	35 patients	Arnica 30X administered orally with sucrose globules. Five globules three times a day (from 2 h before surgery to the last follow-up examination)	Trend towards less postoperative swelling	[46]
	H	Arnica 30X	O	Swelling and pain after cruciate ligament reconstruction	Randomized, double-blind	57 patients	Arnica 30X administered orally with sucrose globules. Five globules three times a day (from 2 h before surgery to the last follow-up examination)	Significant reduction in swelling compared to the placebo	[46]
	H + P	Arnica D6 tablet and 10% *w*/*v A. montana* ethanolic extract ointment	O + T	Pain following carpal tunnel release surgery	Randomized, double-blind	40 patients	D6 tablets from the day of surgery for two weeks and massage with ointment three times daily	Significant pain reduction	[17]
	H	Arnica 30C tablets	O	Pain following tonsillectomy	Randomized, double-blind, placebo-controlled trial	190 patients	Two tablets six times on the first postoperative day and then two twice a day for seven days	Small, but statistically significant, decrease in pain scores	[47]
	H	Arnica 6 CH	SL	Edema, mouth opening, and pain in patients submitted to extraction of impacted third molars	Crossover and double-blind	32 patients	Five drops sublingually three times a day before surgery. Five drops were administered two times a day at the day of surgery, and three times daily the first day after surgery	Significant edema reduction but no effect on mouth opening and pain compared to the control group	[48]
	H	Tablets 30X	SL	Pain, edema, and maximum mouth opening following the extraction of impacted mandibular third molars	Case–control pilot study	23 patients	Four tablets 1 h before the procedure and four tablets × 4 times/day starting 1 h following extraction, and four tablets × 4 times/day for the three following days	Significant reduction in pain, bleeding, bruising, edema, and a decrease in maximum mouth opening compared to the control group	[49]
	H	Arnica 200C	n.s.	Post-extraction pain management in children	Triple-blind randomized controlled trial	44 healthy children (8–12 years old)	Children requiring two clinical sessions of tooth extraction in two different quadrants of the oral cavity received both arnica and ibuprofen with a washout of 10 days	No differences were observed between arnica and ibuprofen	[21]
	H	Arnica capsules	O	Edema and bruising associated with rhinoplasty	Randomized placebo-controlled trial	48 patients	Arnica three times a day for 4 days	Arnica was similar to corticosteroids in reducing edema within 2 days after surgery, with its resolution within 8 days	[50]
Wound healing	H	Arnica 4D	O	Healing of wounds after *hallux valgus* surgery	Randomized double-blinded, parallel-group study	88 patients	10 pills of arnica D4, three times per day	Effectiveness on wound irritation and patient mobility equivalent to diclofenac	[51]
	P	*Arnica* tincture ointment	T	Cicatrization process of aphtha and lesions in human oral mucosa	n.s.	31 patients	*Arnica*-based ointment applied three times per day	Observed anti-inflammatory and wound healing activity on the repair process of lesions in oral mucosa	[52]
	P	Cream containing 5 mg/100 mg *A. montana* extract	T	Reduction in pain associated with arteriovenous fistula puncture	Double-blind single-group randomized clinical trial	71 hemodialysis patients	Before needle insertion, the site was treated for 10 min with 5 mL of cream	Effectiveness in reducing the pain due to arteriovenous fistula puncture	[53]
Trauma-induced backache	H	Arnica 200	O	Lower back pain induced by trauma	Three-arm randomized controlled trial	30 patients	Arnica 200 twice in a day for 15 days and physiotherapy	Significant improvement in pain scores was observed	[54]
Osteoarthritis	P	*A. montana* tincture gel	T	Mild-to-moderate osteoarthritis of the knee	6-week multicenter trial	79 patients	Thin layer of *A. montana* gel applied two times per day	*Arnica* gel was effective and safe	[55]
	P	*A. montana* tincture 50 g/100 g gel (drug-to-extract ratio 1:20)	T	Osteoarthritis of interphalangeal joints of hands	Randomized, double-blind study	204 patients	*Arnica* gel (4 cm strip) was rubbed over the joints thrice daily for 3 weeks	*Arnica* gel was not inferior to ibuprofen when treating osteoarthritis of hands	[56]

Form.: formulation; H: homeopathic; P: phytotherapeutic; Adm.: route of administration; O: oral; T: topical; SL: sublingual; Part.: participants; Ref.: reference; n.s.: not specified.

**Table 4:** Case reports concerning the effectiveness of *Arnica montana* L. formulations.

Use	Form.	Sample/ Extract	Adm.	Treated Condition/ Disease	Study Design	Part.	Treatment	Results	Ref.
Pain and numbness in the hand	H	*Arnica* patch (3X)	T	Cellulitis-derived pain and numbness in the hand	Case report	55-year-old female patient	Patches applied on painful areas of the hand at night for 3 days	Decrease in pain symptoms	[60]
Osteoarthritis	P	200 mg/mL *A. montana* flowering tops in 1:5 herb-to-olive oil ratio	T	Extreme shoulder deterioration due to osteoarthritis	Case report	82-year-old Caucasian female	*Arnica* oil massage followed by acupuncture and therapeutic ultrasound	Decreased pain and partially regained functionality	[61]

Form.: formulation; H: homeopathic; P: phytotherapeutic; Adm.: route of administration; T: topical; Part.: participants; Ref.: reference.

**Table 5:** Clinical trials, observational studies, and other studies concerning the effectiveness of formulations containing *A. montana* L. and other plant extracts.

Use	Form.	Sample/ Extract	Adm.	Treated Condition/ Disease	Study Design	Part.	Results	Refs.
Exercise-induced muscle pain	n.s.	Mud with *A. montana* EO (3%) and menthol (5%)	T	Recovery following a training session	Randomized counterbalanced crossover design	10 resistance-trained men	Enhanced recovery rate of strength and reduced muscle soreness after high-volume exercise	[62]
Arthritis	H	Arnica Comp. -Heel^®^ (“Arnica compositum” ointment, AC, a combined homeopathic ointment containing different plants) + Acidum nitricum (AN) + Hekla lava (HL)	T	Local treatment of symptomatic calcific periarthritis of the shoulder	Pilot study	41 patients	The topical administration of the AC + AN + HL ointment mixture was superior to AC alone	[63]
Postoperative pain	H	Traumeel^®^ S	I	Pain, edema, and trismus associated with the surgical removal of mandibular third molar teeth	Randomized triple-blind clinical trial	17 patients	Obtained results were not different from those of dexamethasone	[64]
	H	Traumeel^®^ S	I, O	Postoperative pain following elective *Hallux valgus* surgery	Triple-arm, quasi-randomized pilot study	30 patients	The tested homeopathic formulation demonstrated efficacy in minimizing postoperative pain	[65]
Seroma reduction	H	*A. montana* C30 and *Bellis perennis* C30	O	Seroma following mastectomy and immediate breast reconstruction	Randomized, double-blind, placebo- controlled trial	55 patients	Reduced seroma formation and opioid intake following mastectomy and reconstruction	[66]
Tendinopathy	H	Traumeel^®^ S	T	Tendinopathies of varying etiology	Nonrandomized, observational study	357 patients	Homeopathic therapy was not inferior to diclofenac as regards pain and motility	[67]
Gonarthrosis	H	Traumeel^®^ S + magnetic field LED therapy	T	Analgesic effect in patients with gonarthrosis	n.s.	90 patients	The combined therapy gave positive results, even if the strongest analgesic factor seemed to be magnetic and LED therapies	[68]
Ankle distortion	P	Combination spray of *Arnica* tincture and hydroxyethyl salicylate	T	Ankle distortion	Randomized double-blind study	570 patients	Significant pain improvement compared to *Arnica* and hydroxyethyl salicylate groups	[69]
Pain related to sports injuries and diseases of the locomotor apparatus	P	Combination spray of *Arnica* tincture and hydroxyethyl salicylate	T	Analgesic effects after the application of electrical pulses for pain production	Randomized, single-blind trial	40 healthy volunteers	The combination preparation was more effective than both the placebo and the individual active agents	[70]
Chronic wounds	P	*A. montana* 0.01% *v*/*w*, *C. officinalis* L., *M. arvensis,* *S. album* powder blend	T	Pain, odor, and exudate in chronic wounds at the end of life	Observational study	50 subjects	Wound care powder for hospice patients with chronic wounds at the end of their life was well tolerated. Significant reduction in pain, odor, and exudate.	[71,72]
Burns	P	Combudoron ^®^ (ethanolic extract of *Urtica urens* and *A. montana*)	T	Efficacy in partial thickness burns	Case report	2 individuals	Beneficial effects on healing of grade 2 laser-induced burns	[73]

Form.: formulation; H: homeopathic; P: phytotherapeutic; Adm.: route of administration; I: injection; O: oral; T: topical; Part.: participants; Ref.: reference; n.s.: not specified.

**Table 6:** *Arnica chamissonis* Less. effectiveness.

Sample/ Extract	Use	Form.	Animal/ In Vitro Model	Treatment	Results	Ref.
*A. chamissonis* ethanolic extract	Oxidative stress	P	In vitro assays	Extracts from flower heads and rhizomes	Chelating power ability, lipid peroxidation, and lipoxygenase inhibitory activity	[36]

Form.: formulation; P: phytotherapeutic.

**Table 7:** Studies with negative outcomes.

Use	Form.	Sample/ Extract	Adm.	Study Design	Treated Condition/ Disease	Treatment	Results	Ref.
Muscle pain and bruising	H	Cream 1X HPUS-7%	T	Randomized, double-blind, placebo-controlled	Bruising and muscle pain	Cream administered to the legs after calf exercise, and 24 and 48 h later	Arnica cream was found to increase leg pain 24 h after calf exercises and any significant results were observed after 48 h	[4]
Postoperative pain and bruising	H	*A. montana* 6X and 30X	OT/ SC	In vivo study	Postoperative pain in cats subjected to a hysterectomy	30 min before surgery and over 72 h 1 mL of *Arnica* 30X per SC route; *Arnica* 30X per OT; *Arnica* 6X OT	Arnica showed less efficacy than ketoprofen and morphine and no differences among *Arnica* formulations and routes of administration were observed	[79]
	H	Arnica tablets 30C or 6C	OT	Double-blind, placebo-controlled, randomized trial	Pain and bruising in 64 adults undergoing surgery for carpal tunnel syndrome	Three tablets daily of arnica 30C or 6C or the placebo for 7 days before and 14 days after surgery	No significant effects were observed in reducing postoperative pain, bruising, and swelling compared to the placebo	[80]
Knee ligament reconstruction	H	Complex containing *A. montana* 5 CH, *B. alba* 5 CH, *H. perforatum* 5 CH, and *R. graveolens* 3 DH	O	Phase III randomized placebo-controlled study	Ability to reduce morphine consumption after knee ligament reconstruction	Five granules were assumed from days 0 to 4 and added to other analgesic treatments	The activity was not superior to the placebo	[81]
Chronic neck pain	P	*A. montana* massage oil	T	Randomized controlled trial	Chronic pain	12 weeks of a partner-delivered home-based cupping massage using *A. montana* massage oil, compared to the same period of progressive muscle relaxation	Cupping massage was not more effective than progressive muscle relaxation in reducing chronic non-specific neck pain	[82]
Muscle soreness	H	Arnica 6X pellets	SL	Double-blind, randomized study	Delayed onset muscle soreness	23 participants aged between 18 and 25 were instructed to take three pellets sublingually before the bench stepping	Arnica 6X was not an efficacious agent for moderating DOMS	[24]

Form.: formulation; H: homeopathic; P: phytotherapeutic; Adm.: route of administration; O: oral; T: topical; OT: oral transmucosal; SC: subcutaneous; SL: sublingual; Ref.: reference.

## Data Availability

Not applicable.

## Abbreviations

Adm.	route of administration
AP-1	activator protein-1 transcription factor
CAT	catalase
COX-2	cyclooxygenase-2
DOMS	delayed onset muscle soreness
Form.	formulation
GSH	glutathione
H	homeopathic
ICAM-1	intercellular adhesion molecule
IFN-γ	interferon gamma
IL-12	interleukin-12
IL-1β	interleukin -1-beta
IL-6	interleukin-6
MCP-1	monocyte chemoattractant protein-1
MMP1	collagenase-1
MMP13	interstitial collagenase-3
NF-kB	transcription factor nuclear factor kappa B
O	oral
OT	oral transmucosal
P	phytotherapeutic
ROS	reactive oxygen species
SC	subcutaneous
SOD	superoxide dismutase
STLs	sesquiterpene lactones
T	topical
TNFα	tumor necrosis factor alpha
UHPLC-HRMS	ultra-high-performance liquid chromatography with high-resolution mass spectrometry
UV	ultraviolet
